# Advances of naturally derived biomedical polymers in tissue engineering

**DOI:** 10.3389/fchem.2024.1469183

**Published:** 2024-11-20

**Authors:** Tao Hu, Jie Fang, Yang Shen, Mingyang Li, Bin Wang, Zushun Xu, Weikang Hu

**Affiliations:** ^1^ Ministry of Education Key Laboratory of the Green Preparation and Application for Functional Materials, Hubei Key Laboratory of Polymer Materials, School of Materials Science and Engineering, Hubei University, Wuhan, China; ^2^ Shenzhen Youcare Medical Equipment Co. Ltd., Shenzhen, China; ^3^ Department of General Surgery, Shenzhen Children’s Hospital, Shenzhen, China

**Keywords:** natural polymers, tissue engineering, biomedical application, scaffolds, regenerative medicine

## Abstract

The extensive utilization of natural polymers in tissue engineering is attributed to their excellent biocompatibility, degradability, and resemblance to the natural extracellular matrix. These polymers have a wide range of applications such as delivering therapeutic medicine, detecting diseases, sensing biological substances, promoting tissue regeneration, and treating diseases. This is a brief review of current developments in the properties and uses of widely used biomedical polymers derived from nature. Additionally, it explores the correlation between the characteristics and functions of these materials in different biomedical applications and highlights the prospective direction for the advancement of natural polymer materials in tissue engineering.

## 1 Introduction

Biomedical polymers have played a crucial role in the development of tissue engineering, which aims to create functional biological substitutes to restore, maintain, or improve damaged tissues or organs (Lee et al., 2014). Biomedical polymers have unique properties that make them suitable for various applications in tissue engineering, such as flexibility, biocompatibility, and mechanical strength (Khan et al., 2023). One of the most essential properties of those polymers are biodegradable, which means they can be degraded and even absorbed by the body over time (Song et al., 2018). This property is critical for temporary scaffolds or implants that are designed to promote tissue growth before gradually degrading as the new tissue forms and matures. The polymer degradation rate can be tailored to match the rate of tissue regeneration, resulting in a smooth transition from the artificial scaffold to the natural extracellular matrix (Echeverria Molina et al., 2021). Furthermore, these polymers can be fabricated into versatile materials that can mimic the structure and function of native tissues or can be designed into diverse microstructures to attain specific performance objectives, and they are commonly employed as a supportive substrate to transport cells and therapeutic agents to a particular location (Zarrintaj et al., 2023). Thus, biomedical polymers are used in various areas such as tissue engineering, drug delivery systems, biosensors, regenerative medicine, artificial organs, hemodialysis, osseointegration, and bone injury repair (Kurowiak et al., 2023).

Biomedical polymers can be natural or synthetic, the main difference between these two types of polymers is their structure (Prete et al., 2023). Natural polymers, such as proteins and polysaccharides, have the ability to fold into complex shapes without assistance. The specific sequence of amino acids in proteins or the composition and linkages of monosaccharides in polysaccharides determine their fundamental structures, which in turn, define their biological functions. (Bealer et al., 2020; Chen et al., 2022). Naturally derived polymers have several advantages including biocompatibility, biodegradability, biomimicry, and the potential for modification (Prete et al., 2023; Satchanska et al., 2024). Natural polymers like collagen, gelatin, chitosan, and hyaluronic acid, which are derived from biological sources, exhibit excellent biocompatibility. This means they do not cause inflammation or elicit a strong immune response when implanted in the body, unlike some synthetic materials. Most natural polymers are enzymatically degradable in physiological conditions, allowing them to be broken down and resorbed by the body over time as new tissue forms (Song et al., 2018). This characteristic is advantageous for tissue engineering scaffolds where the material should degrade at a rate matching new tissue formation. In terms of biomimicry, natural polymers like collagen, elastin, and glycosaminoglycans closely mimic the cellular microenvironment of native tissues, providing appropriate biological cues to guide cell adhesion, proliferation, and differentiation (Gomez-Florit et al., 2020). Additionally, natural polymers can be chemically modified to adjust their mechanical properties and degradation kinetics (Demirgöz et al., 2000), as well as incorporate growth factors, drugs, or enable crosslinking, offering a high degree of customization (Pires et al., 2023).

In contrast, most synthetic polymers (such as polyesters, poly (ethylene glycol), and polycarbonates) have simpler, more random structures. Furthermore, naturally derived polymers typically degrade in the environment and interact well with biological entities such as cells and tissues (Geevarghese et al., 2022). However, they have some drawbacks, such as poor mechanical properties, uncontrollable decomposition, and the risk of causing the body’s immune system to react negatively, limiting their use in biological organisms (Xia et al., 2022). Synthetic polymers, on the other hand, have good controllability in terms of composition, structure, mechanical properties, and degradation behavior, making them a promising avenue for biomedical applications despite their lack of inherent biological activity (Reddy et al., 2021). As a result, it is critical to maximize the benefits of both natural and synthetic polymers in biomedical fields.

Biomedical polymers can be synthesized or modified with a wide range of physical and chemical properties to suit different tissue engineering applications (Wei et al., 2018). For example, polymers used for bone tissue engineering should have high mechanical strength and stiffness to withstand the loads and stresses experienced by bones (Bakhtiari et al., 2023). On the other hand, polymers for soft tissue engineering, such as skin or blood vessels, require flexibility and elasticity to mimic the natural behavior of these tissues (Aurand et al., 2012; Coenen et al., 2018). The porosity and surface properties of biomedical polymers play a significant role in cell adhesion, proliferation, and differentiation. Porous polymeric scaffolds provide a three-dimensional environment for cells to grow and organize into functional tissues (Loh and Choong, 2013; Lutzweiler et al., 2020). The pore size, interconnectivity, and overall porosity can be controlled to facilitate cell migration, nutrient transport, and vascularization (Murphy and O'Brien, 2010; Somo et al., 2015). Surface modifications, such as protein coatings or chemical functionalization, can further enhance cell-material interactions and guide the formation of tissues (Kim et al., 2021; Hu et al., 2023; Nitti et al., 2023).

Biomedical polymers can be manipulated into different forms and configurations through methods such as electrospinning, 3D printing, or solvent casting (Stratton et al., 2016; Ammann et al., 2019). The ability to adapt and change allows for the creation of intricate support structures with accurate shapes and designs that closely mimic the original tissue (Liu et al., 2023). The capacity to tailor the form and composition of polymeric scaffolds is especially advantageous for fabricating implants or tissue constructs that are specific to individual patients (Bertsch et al., 2023; Ramezani and Mohd Ripin, 2023; Suamte et al., 2023).

**FIGURE 1:**
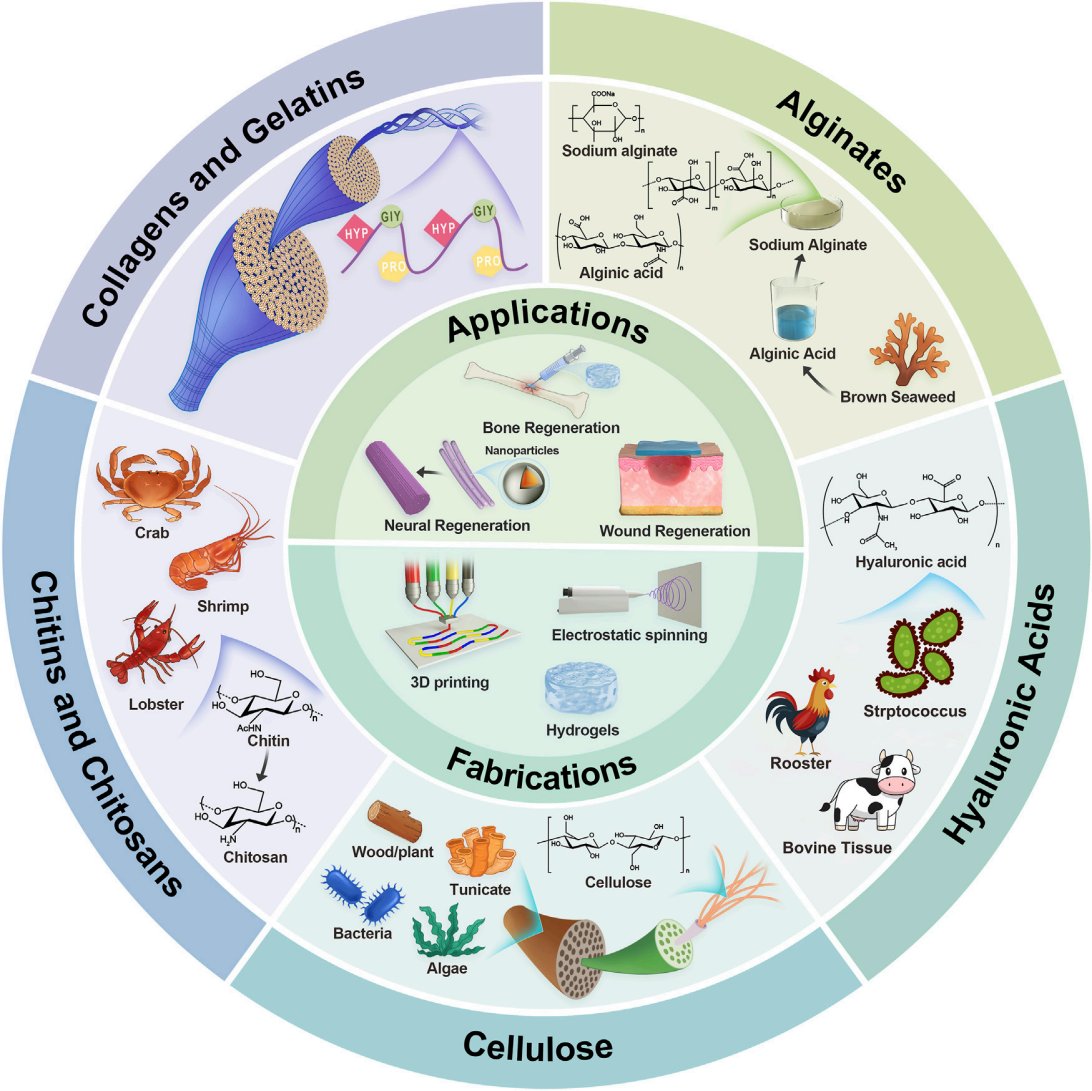
Illustrative schematic of naturally derived polymers’ origin, structures, fabrications, and applications. The next section will explore the characteristics of each polymer in tissue engineering.

This review provides an overview of the most widely used naturally derived biomedical polymers in tissue engineering, elucidating their chemical compositions, physical and chemical characteristics, and biological functions. It also presents the most recent research discoveries in the biomedical domain (Figure 1). In addition, the article provides a detailed introduction to various novel medical polymer materials, including their developmental context, key features, and potential uses in the field of medicine. Ultimately, the review discusses the challenges addressed by biopolymers and proposes prospective insights on future research directions.

## 2 Naturally derived polymers and their derivatives in biomedical applications

### 2.1 Collagens and gelatins

Collagen and gelatin are proteinaceous substances that originate from animal sources, specifically the skin, bones, and connective tissues of cows, pigs, and fish (Gómez-Guillén et al., 2011; Nurilmala et al., 2022). Collagen is the primary protein that provides structure to the body’s connective tissues, while gelatin is a substance derived from collagen (Matinong et al., 2022). Collagen and gelatin are both biocompatible and biodegradable, which makes them especially suitable for a wide range of biomedical applications. Their distinct characteristics, including their ability to provide structural support, form gels, and have little chance of causing an immune response, make them highly valuable in various medical fields such as tissue engineering, wound healing, drug delivery, and other medical applications (Gómez-Guillén et al., 2011; Nurilmala et al., 2022).

Collagen is the predominant protein found in mammals, comprising around 25%–35% of the total protein composition in the body. It is present in connective tissues such as cartilage, bones, tendons, ligaments, and skin, as well as in corneas, blood vessels, the gut, intervertebral discs, and teeth (Shenoy et al., 2022). Collagen is a resilient and indissoluble protein composed of amino acids that arrange themselves into a triple helix structure known as a collagen helix. Collagen is responsible for providing structural support and is essential for preserving the strength and flexibility of different tissues. Collagen contributes to the fortification of bones, facilitates the elasticity of skin and tendons, and aids in the process of recovery following an accident. The human organism contains a total of 29 different forms of collagen, with types I, II, and III constituting around 80%–90% of the overall collagen content. Type I collagen is the most prevalent in the skin, accounting for 80% of the collagen there, and type III collagen corresponds to around 15%. On the other hand, type II collagen is the primary component of the extracellular matrix of cartilage, making up 90%–95% of the collagen found there (Campos et al., 2023). It is frequently employed in cosmetic items, supplements, and medicinal uses like as wound healing and tissue regeneration.

Gelatin is a protein-rich substance derived from the skins of pigs and cows, as well as demineralized animal bones. It is frequently found in various food products such as gummy candies, marshmallows, and pastilles. Gelatin is created through a carefully controlled process that involves heat and acid or alkaline treatment. It is derived from animal sources by partially hydrolyzing collagen (Mikhailov, 2023). It is commonly utilized as a gelling agent in various food products, including jellies, desserts, candies, and certain dairy items. Gelatin is widely used in various industries, including pharmaceuticals, photography, and the manufacturing of capsules and coatings.

The FDA approves collagens and gelatins for use in various medical products, including wound dressings, hemostatic agents, tissue sealants, and dermal fillers (Zhang and Wang, 2024). These versatile biomaterials provide a three-dimensional environment for cell growth and tissue regeneration. They can be combined with stem cells, growth factors, and other bioactive molecules to enhance the healing process (Sowbhagya et al., 2024; Zhu et al., 2024). Collagen-based scaffolds and matrices are utilized in clinical settings for tissue engineering and regenerative medicine applications (Dong and Lv, 2016), these scaffolds have a wide range of applications, including the regeneration of skin, bone, cartilage, tendons, and nerves, as well as in drug and gene delivery systems. For instance, gelatin-based hydrogels are being explored in clinical trials as injectable carriers for cells and drugs, allowing for sustained and localized delivery of medications, proteins, and nucleic acids (Jia et al., 2024). Additionally, collagen and gelatin nanoparticles enable targeted delivery to specific tissues or cell types. Moreover, collagen and gelatin-based bioinks facilitate the 3D printing of complex tissue constructs with high shape fidelity (Yang et al., 2024). They can be combined with living cells to create patient-specific tissues and organs. These materials enhance the biocompatibility and cell adhesion of medical implants and devices, and they can incorporate antimicrobial agents to prevent infections associated with implants. Furthermore, collagen and gelatin-based 3D matrices mimic the native tissue microenvironment, allowing for the development of more physiologically relevant *in vitro* models for diseases such as cancer and fibrosis (Dong et al., 2024). Table 1 summarizes recent studies on collagen and gelatin and their outcomes in tissue engineering and regenerative medicine.

**TABLE 1:** Recent studies on collagen and gelatin and their outcomes.

Collagen/Gelatin type	Research topic	Research outcome	References
Type I Collagen	Tissue engineering	Collagen hydrogel systems for tissue engineering and regenerative medicine	Leonard et al. (2024)
Type II Collagen	Cartilage repair	collagen scaffolds repair critical-sized osteochondral defects	Hu et al. (2024)
Gelatin	Cardiac tissue engineering/Bone tissue engineering	Gelatin-based hybrid scaffolds in cardiac tissue engineering/nanocomposite biomaterials based on gelatin for bone tissue engineering	Asl et al. (2024), Salehi Abar et al. (2024)
Recombinant Human Collagen	Tissue engineering	Various biomaterials in biomedicine, particularly in tissue engineering and regenerative medicine	Cao et al. (2024)

Collagen and gelatin are ideal materials for skin regeneration and wound healing because of their high biocompatibility (Kang and Park, 2021; Gajbhiye and Wairkar, 2022). Collagen is the skin’s principal structural protein, and gelatin is formed from partially hydrolyzed collagen. Because they are both naturally occurring chemicals in the human body, they are biocompatible with human tissues and do not elicit strong immune reactions. Furthermore, because collagen and gelatin form a porous three-dimensional network structure, these polymers can operate as a scaffold for cells, allowing for controlled release of bioactive chemicals such as growth factors and promoting cell proliferation and tissue repair. The human body’s enzymes, such as collagenase, may degrade both collagen and gelatin. The products of degradation can be digested and absorbed by the body, which aids in tissue regeneration and repair. To summarize, collagen and gelatin have excellent biocompatibility, biodegradability, and cell affinity, allowing them to provide an optimal three-dimensional environment for cell adhesion, migration, and proliferation.

One of the most commonly used materials based on collagen and gelatin derivatives is GelMA (Gelatin methacryloy) (Yue et al., 2015). GelMA is a chemically synthesized polymer formed from gelatin, which is obtained by breaking down collagen through hydrolysis. GelMA has exceptional biocompatibility and biodegradability, rendering it well-suited for tissue engineering endeavors that necessitate the eventual replacement of the scaffold or construct by the body’s own cells and tissues.

**FIGURE 2:**
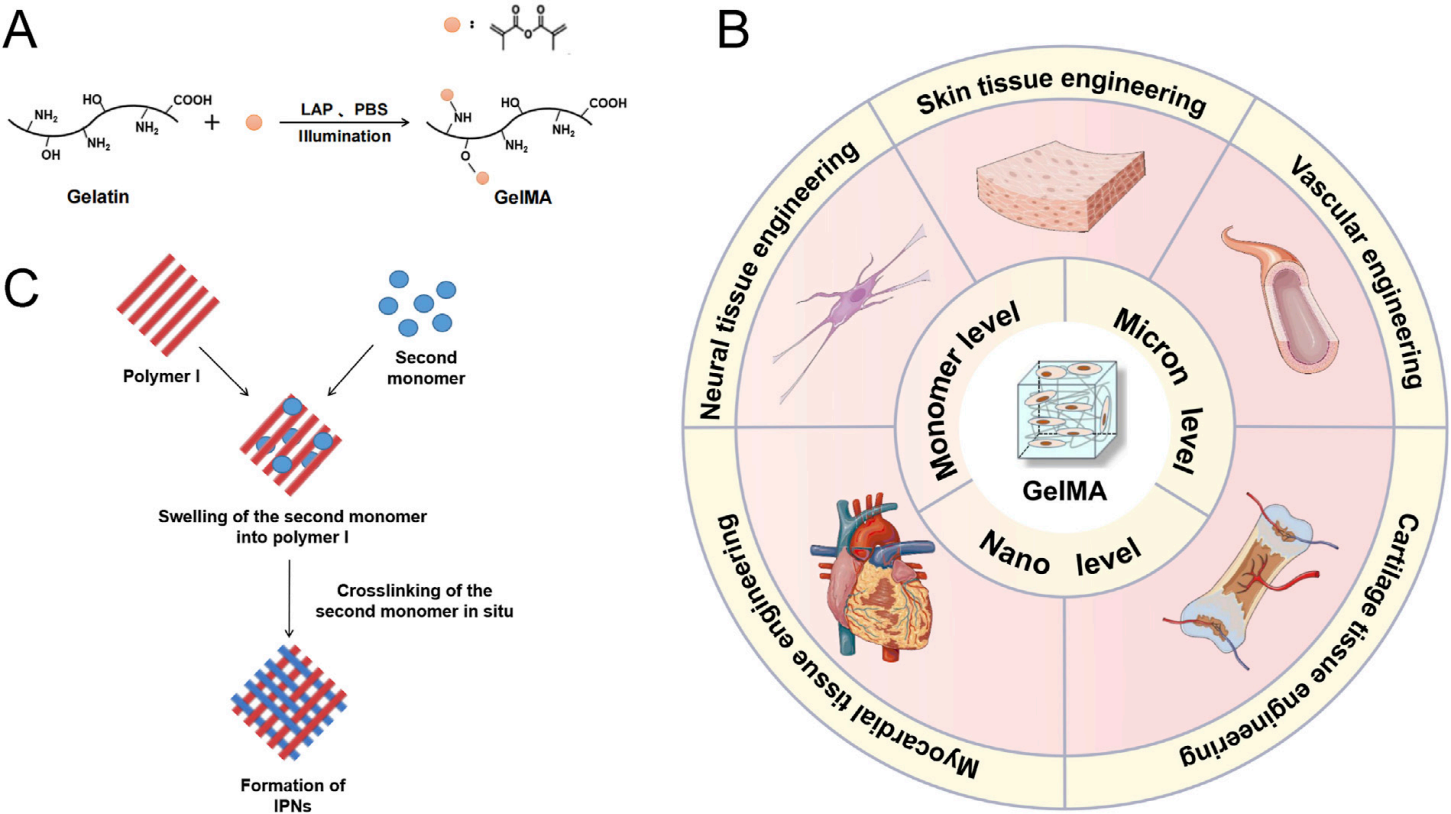
**(A)** Diagram illustrating the process of GelMA formation from gelatin through a substitution reaction. **(B)** GelMA Hydrogel: Methods of Modification and Applications in Tissue Engineering. Schematic representation of the formation of interpenetrating polymer networks (IPNs). **(C)** Diagram illustrating the process of Interpenetrating Polymer Networks (IPNs) formation. Reproduced with permission from the ref. Guo et al. (2024), Copyright 2024, Elsevier. (This work is licensed under CC BY-NC-ND 4.0. To view a copy of this license, visit https://creativecommons.org/licenses/by-nc-nd/4.0/).

GelMA is widely used in tissue engineering research and applications due to its biocompatibility, adjustable mechanical properties, capacity to be crosslinked by light, support for cell adhesion, adaptability, and ability to be printed. GelMA is always fabricated for hydrogels in tissue engineering (Piao et al., 2021). GelMA has methacryloyl groups that can undergo photocrosslinking when exposed to ultraviolet (UV) or visible light with the help of suitable photoinitiators. The photocrosslinking approach enables the creation of hydrogels in their original location with precise control over time and space. This process allows for the entrapment of cells and bioactive compounds within the hydrogel matrix. Besides, by changing the polymer concentration, the degree of methacryloyl substitution, and the crosslinking density, researchers may readily vary the mechanical characteristics of GelMA hydrogels. The GelMA hydrogels have the capacity to adjust the mechanical properties of different native tissues enables researchers to replicate them accurately, hence facilitating the desired cellular response and tissue development (Guo et al., 2024) (Figure 2). GelMA contains cell-binding motifs, including RGD (Arginine-Glycine-Aspartic acid) sequences, that enhance cell adhesion and proliferation. These patterns are crucial for tissue engineering applications because they enable interactions between cells and the surrounding matrix, and they also aid in cellular processes such as migration, differentiation, and tissue creation. GelMA has versatility since it can be subjected to modifications involving diverse functional groups. This enables the integration of supplementary bioactive molecules, such growth factors, medicines, or other biomolecules. Researchers can utilize this adaptability to develop personalized hydrogel systems designed for precise tissue engineering purposes. Furthermore, GelMA hydrogels are suitable for a range of biofabrication methods, such as bioprinting, since they can be extruded and maintain their structural integrity. This allows for the development of intricate 3D tissue structures.

GelMA hydrogels are versatile molecular carriers in tissue engineering, as various particles including nanoparticles, bioactive molecules, cells, extracellular vesicles, and cytokines could be encapsulated inside, which realizes the controlled and sustained release of these biomolecules and therapeutic agents. This encapsulation capability allows for the creation of instructive microenvironments that can modulate cellular behavior, promote tissue regeneration, and enhance therapeutic efficacy.

**FIGURE 3:**
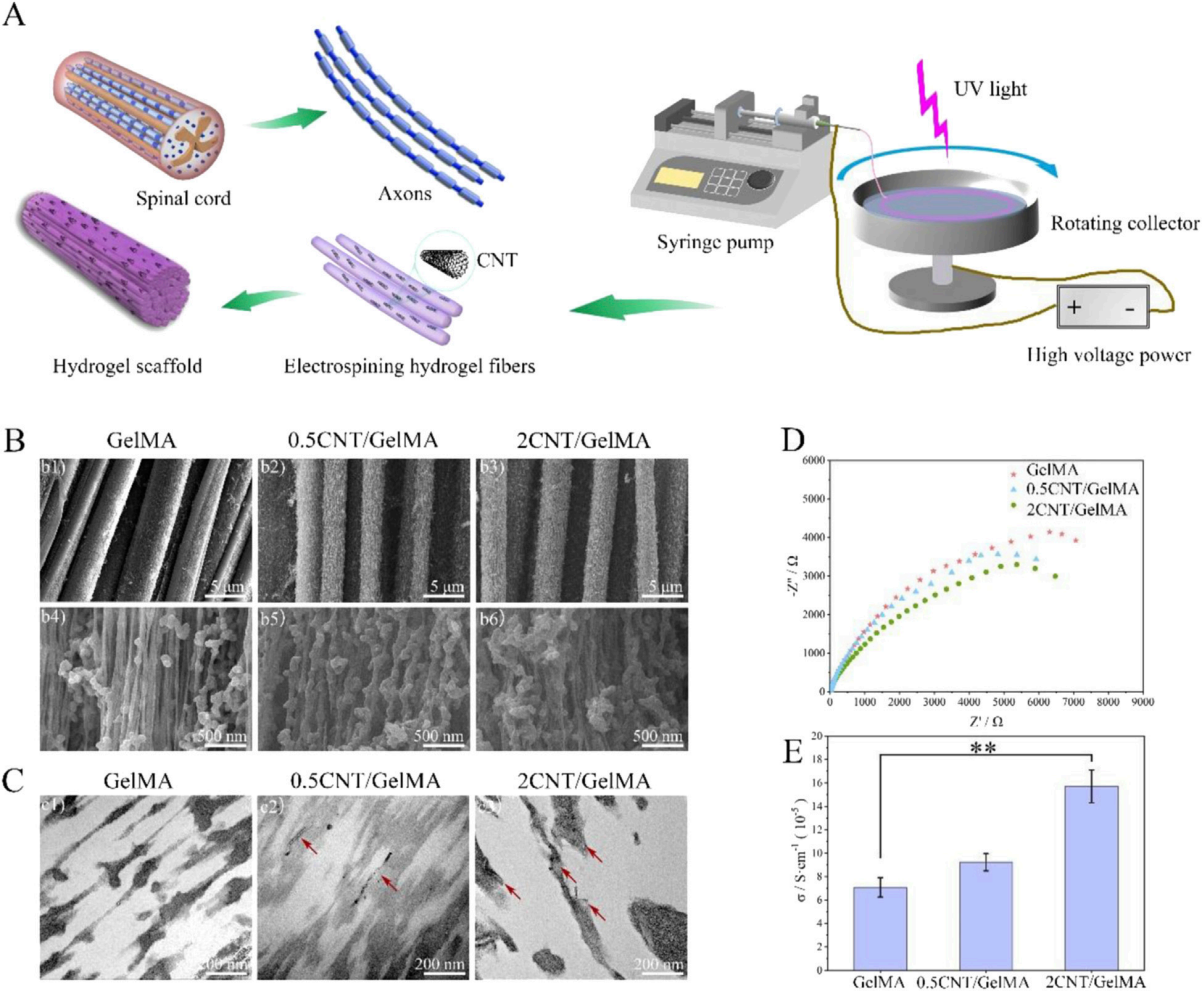
CNT/GelMA-directed conductive hydrogel fiber characterization. **(A)** Spinal cord and axon-inspired CNT/GelMA hydrogel fiber schematic. **(B)** Micron-scale SEM fiber pictures. **(C)** GelMA longitudinal CNT TEM images (red arrows). **(D,E)** Hydrogel fiber electrical conductivity. (**p < 0.01). Reproduced with permission from the ref. Yao et al. (2024), Copyright 2024, Elsevier. (This work is licensed under CC BY-NC-ND 4.0. To view a copy of this license, visit https://creativecommons.org/licenses/by-nc-nd/4.0/).

For example, Yao et al. applied by electrospun carbon nanotubes (CNTs) into gelatin methacrylate (GelMA) hydrogels to create aligned conductive threads (Figure 3). These CNT/GelMA fibers mirrored neural axons’ aligned organization, conductivity, and soft mechanical characteristics. Electrical stimulation (ES) increased neural cell proliferation, aligned adhesion, neuronal differentiation, and neurite sprouting *in vitro*. CNT/GelMA fibers were implanted at the injury site for *in vivo* testing in rats. The aligned fiber arrangement regenerated neural fibers, and conductive CNTs increased tissue conductivity as GelMA degraded. Behavioral and electrophysiological tests showed that aligned conductive fibers and ES restored motor function, remyelination, and axonal regeneration in rats. Electroconductive hydrogel fibers combined scaffold regenerative medicine with electrical stimulation, promising spinal cord damage repair (Yao et al., 2024).

**FIGURE 4:**
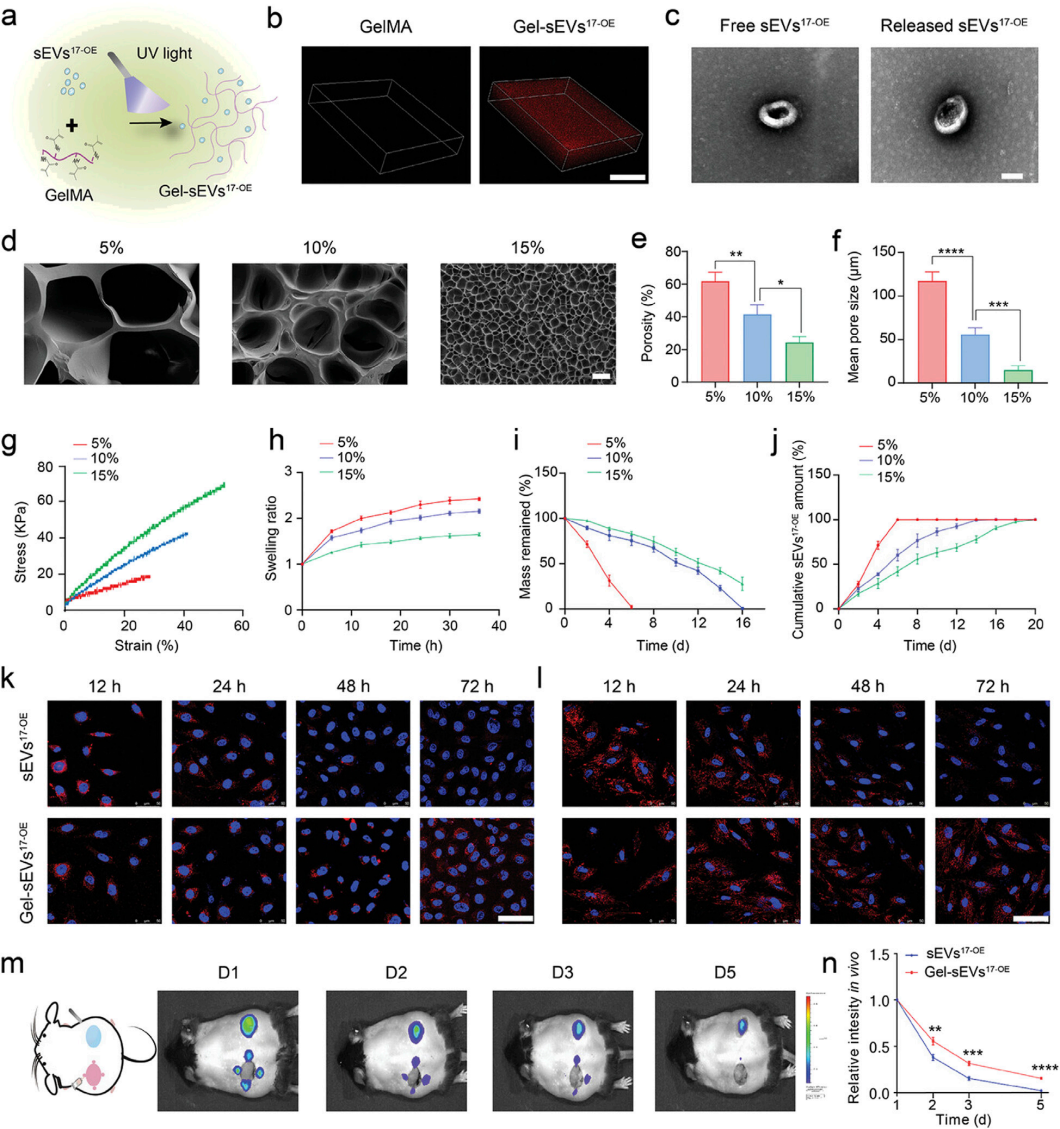
GelMA hydrogel loaded with sEV17-OE was fabricated and characterized. **(A)** Gel-sEVs17-OE preparation schematic diagram. **(B)** CLSM images show the distribution of sEVs17-OE in GelMA hydrogel. **(C)** Typical TEM images of free and released sEVs17-OE from GelMA hydrogel (scale bar = 100 nm). **(D)** Cross-section SEM images of GelMA hydrogels at 5%, 10%, and 15% concentrations (scale bar = 20 µm). **(E–I)** Porosity, mean pore size, stress-strain curves, swelling ratio, and degradation of GelMA hydrogels at 5%, 10%, and 15% concentrations (n = 3 per group). **(J)** Cumulative release profile of sEVs17-OE from GelMA hydrogel at various concentrations over 20 days (n = 3 per group). **(K, L)** Typical fluorescent images of HG-HUVECs and HG-HDFs co-incubated with equivalent DiI-stained sEVs17-OE (red) in free form or released from GelMA hydrogel, with nuclei labeled with Hoechst (blue). Scale bar: 50 µm. **(M)** Typical *in vivo* imaging images demonstrating the retention of DiI-stained sEVs17-OE in free form and GelMA encapsulation on wounds at the specified time point. **(N)** Quantitative results of released sEVs17-OE intensity *in vivo* (m) (n = 3 per group). The data is shown as the mean ± SD. One-way ANOVA was used to compare the groups, followed by Tukey’s posttest. *p < 0.05, **p < 0.01, ***p < 0.001, ****p < 0.0001, ns = not significant compared to the indicated group. Reproduced with permission from the ref. Wei et al. (2024), Copyright 2024, Wiley. (This work is licensed under CC BY 4.0. To view a copy of this license, visit https://creativecommons.org/licenses/by/4.0/).

MiR-17-5p-engineered sEVs (sEVs17-OE) in a GelMA hydrogel were created by Wei et al. for diabetic wound therapy (Figure 4). From lentivirus-transfected hucMSCs with miR-17–5p, SEVs17-OE were identified. After characterisation, sEVs17-OE were introduced to HG-induced human umbilical vein endothelial cells (HG-HUVECs) and human dermal fibroblasts. HG-HUVECs and HG-HDFs were affected by sEVs17-OE, and the mechanism was investigated. In conclusion, a new bioactive wound dressing was made using sEVs17-OE-loaded GelMA hydrogel. Targeting p21 and phosphatase and tensin homolog (PTEN) optimized HG-HUVECs and HG-HDFs *in vitro*. *In vivo*, Gel-sEVs17-OE improved diabetic wound healing by increasing local angiogenesis and collagen deposition (Wei et al., 2024).

Sun et al. create gelatin methacryloyl (GelMA), a cell-affine hydrogel scaffold, from gelatin. GelMA is blended with other components to produce a bi-layer porous hydrogel scaffold with varying modulus and composition at the upper and bottom levels using 3D printing. Black phosphorus (BP) and hUMSC exosomes (exos) make GelMA in the upper scaffold less elastic and better for BMSC cartilage differentiation. GelMA in the lower scaffold contains β-tricalcium phosphate (β-TCP), which aids bone formation and repair, together with BP and hUMSCs exos. The inclusion of β-TCP improves the hydrogel scaffold’s elastic modulus, promoting osteogenic development of BMSCs. *In vitro* research reveals that bi-layer scaffolds increase osteogenesis and chondrogenic differentiation. MRI and micro-CT data reveal that the 3D-printed bi-layer GelMA composite scaffold repairs rabbit cartilage-bone injuries near normal tissue (Sun et al., 2024).

**FIGURE 5:**
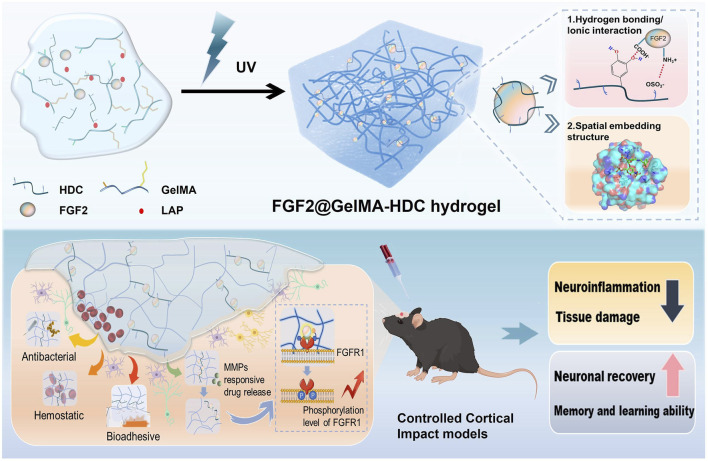
Schematic of FGF2@GelMA-HDC hydrogel production for controlled cortical impact models and brain tissue regeneration. Reproduced with permission from the ref. Shen et al. (2024), Copyright 2024, Elsevier. (This work is licensed under CC BY-NC-ND 4.0. To view a copy of this license, visit https://creativecommons.org/licenses/by-nc-nd/4.0/).

GelMA-HDC, a multifunctional growth factor-affinity system, was developed by Shen et al. After an injury such as TBI (Traumatic brain injury), this system can deliver growth factors to protect and repair the brain (Figure 5). GelMA-HDC, an injectable hydrogel comprising gelatin-methacryloyl (GelMA) and heparin-dopamine conjugate (HDC), has smaller pores, greater adhesion, and softer mechanical qualities than GelMA. FGF2 stability and regulated release in brain lesions were improved by GelMA-HDC. FGF2-HDC complexes improved the FGF2-FGFR1 signaling pathway, providing strong neuroprotection. GelMA-HDC hydrogels at the damage site in TBI mice also demonstrated healing capability. The outcomes included mending injured cortical structures, lowering neuroinflammation, preserving neurons, and improving mouse learning and memory. The study reveals the first results on treating TBI with mussel-inspired GelMA-based hydrogels that bind FGFs well. This novel strategy to using heparin-binding FGFs to treat neurological illnesses like TBI has great potential (Shen et al., 2024).

Liang et al. created a physically cross-linked hydrogel precursor from gelatin methacryloyl (GelMA), poly (thioctic acid) (P (TA)), PAAc/ACP, PA, and an FDA-approved photoinitiator solution. Visible light initiation created a chemically and physically cross-linked hybrid hydrogel with molecular flexibility at physiological temperature. Mechanical parameters of the GelMA/P (TA)/PA hydrogel included 206% elongation at break and 459 kPa compressive strength at 90% strain. The optimised visible-light cross-linked hydrogel may attain lap-shear adhesion strength above 100 kPa to gelatin after 2 min of irradiation. Biodegradable and biocompatible, this hydrogel may adhere to aquatic tissue surfaces for lengthy periods. Even after a month in PBS, it sticks on the intestine. *In vivo* liver injury models showed that the hydrogel was hemostatic and had healing potential. These data suggest that this safe *in situ* injection technique and polymer system could help tissue repair and regeneration, especially in non-compressive wound repair (Liang et al., 2024).

In summary, collagens and gelatins have a proven clinical track record and are actively being researched for various applications in tissue engineering, drug delivery, 3D bioprinting, surface modification, and disease modeling. Ongoing research seeks to further expand and optimize their use in regenerative medicine and other biomedical fields. At present, there are still several challenges using collagen and gelatin in tissue engineering. One of the essential issues regarding natural sources of collagen and gelatin, such as bovine or porcine tissues, is that they can cause batch-to-batch changes in characteristics and composition, which influence the quality of products. Besides, collagen and gelatin usually have weak mechanical qualities as compared to native tissues, particularly in terms of strength and stiffness. Collagen and gelatin are biodegradable. However, their disintegration rate may not match new tissue development, causing premature structural damage or inadequate tissue regeneration. Collagen and gelatin are biocompatible, although some people may have immunological responses to them, especially if they come from non-human origins or are processed chemically. Collagen and gelatin support cell adhesion and proliferation, however differentiation and matrix synthesis may be limited (Huang et al., 2023). Despite these obstacles, collagen and gelatin are intriguing tissue engineering materials. Research is underway to overcome these biomaterials’ limitations and use them for tissue engineering.

### 2.2 Chitins and chitosans

Chitin and chitosan, obtained from the outer shells of crustaceans and the cellular walls of fungi, exhibit numerous favorable traits that render them appealing biomaterials for tissue engineering applications (Elieh-Ali-Komi and Hamblin, 2016). Chitin and chitosan are biocompatible polysaccharides, indicating that they are generally not poisonous and do not cause immune responses, which makes them appropriate for use in biomedical applications (Baharlouei and Rahman, 2022). These items are capable of undergoing biodegradation, which means they can be broken down and replaced by new tissue over a period of time in a controlled manner. Chitin and chitosan have the ability to undergo shape transformation, resulting in various forms such as hydrogels, sponges, nanofibers, and scaffolds (Oryan and Sahvieh, 2017; Islam et al., 2020). This enables the creation of three-dimensional structures that closely resemble the extracellular matrix (ECM) found in actual tissues. These biomaterials possess appropriate mechanical properties, porosity, and surface features that can be customized to meet the unique needs of different tissue types. Chitosan, specifically, demonstrates antibacterial characteristics that aid in the prevention of bacterial and fungal infections in tissue engineering applications (Teixeira-Santos et al., 2021). Researches have shown that both chitin and chitosan promote the attachment, growth, and specialization of cells, therefore facilitating the regeneration and restoration of tissues (Jayakumar et al., 2011; Kim et al., 2023). Chitin and chitosan have the ability to transport various bioactive substances, including as medications, growth factors, and genes (Baharlouei and Rahman, 2022; Desai et al., 2023). Furthermore, biomaterials derived from chitin and chitosan have shown promise in facilitating the process of wound healing and regenerating many types of tissues, including as bone, cartilage, skin, and nerve tissue, in the field of tissue engineering (Kim et al., 2023; Elizalde-Cárdenas et al., 2024). Their capacity to facilitate cell adhesion, proliferation, and specialization, along with their ability to control bleeding and combat microbial infections, enhance their effectiveness in various uses.

Chitosan is a biopolymer composed of β-(1–4)-linked D-glucosamine and N-acetyl-D-glucosamine, which are generated from chitin. Chitosan is widely utilized in tissue engineering, particularly in the areas of wound healing and drug delivery. The substance can be utilized either in its raw form or subjected to further processing to broaden its scope of potential uses. Chitosan and its derivatives have the ability to generate fibers, hydrogels, and sponges, which has led to an increase in research and studies on this adaptable biopolymer (Elizalde-Cárdenas et al., 2024). The results of recent research on chitins and chitosans in tissue engineering and regenerative medicine are compiled in Table 2.

**TABLE 2:** Recent studies on chitins and chitosans and their outcomes.

Chitin/Chitosan type	Research topic	Research outcome	References
Chitosan	Chitosan-based hydrogels	Developed chitosan hydrogels for sustainable tissue engineering	Mishra et al. (2024)
Chitosan	Antimicrobial activity	Chitosan nanoparticles extraction	Sathiyabama et al. (2024)
Chitin	Chitin-based materials for wound healing	Supramolecular chitin-based hydrogels with self-adapting and fast-degradation properties	Shi et al. (2024)
Chitosan	Chitosan as a vaccine adjuvant	Reviewed chitosan’s potential as a vaccine adjuvant, demonstrating its ability to enhance immune responses	Kumar et al. (2024)
Chitosan	Chitosan-based films for packaging	Investigated chitosan-based films for packaging	Mohammed et al. (2024)
Chitosan	Chitosan-based materials for bone tissue engineering	Investigated chitosan-based composites for bone tissue engineering, demonstrating enhanced properties for promoting growth	Babu et al. (2024), Purohit et al. (2024)

Sakai et al. investigated the fabrication of wound dressings by employing hydrogel precursor inks that consisted of phenolated chitosan (chitosan-Ph) and chitosan nanofibers (chitosan-NF) (Sakai et al., 2024). The 3D printing process involved the use of horseradish peroxidase (HRP) to induce hydrogelation. Through the manipulation of chitosan-Ph and chitosan-NFs composition, they were able to regulate the viscosity of the ink, the time it takes for gelation to occur, and the stiffness of the hydrogel. The composite hydrogel exhibited antibacterial characteristics and shown compatibility with cells. More precisely, hydrogels containing 2% (w/w) chitosan-Ph and 1% (w/w) chitosan-NFs exhibited superior precision in design and showed promising capabilities for healing wounds when compared to commercially available dressings. The hydrogels facilitated the process of re-epithelialization, development of granulation tissue, and structure of the extracellular matrix, without exacerbating inflammation or foreign body reaction. The results emphasize the efficacy of integrating chitosan-NF into chitosan-Ph inks to produce customized wound dressings using extrusion 3D printing, signifying a notable progress in personalized wound treatment.

Cai et al. produced a composite hydrogel named MAC-PEG-DMA, comprising methacrylimide-chitosan (MAC), 4-arm polyethylene glycol (PEG), and dopamine (DMA) (Cai et al., 2024). The hydrogel was synthesized using a one-pot photo-crosslinking technique and included with A-chain homodimers of platelet-derived growth factor (PDGF-AA). The hydrogel that was produced exhibited enhanced mechanical strength and hemostatic capabilities, as evidenced by both *in vitro* blood clotting assay and rat liver hemorrhage assay. The hydrogel loaded with PDGF-AA was seen to expedite cell migration and proliferation through the gradual release of PDGF-AA, hence successfully promoting wound healing. Analysis of skin wounds treated with this hybrid hydrogel revealed accelerated wound healing and enhanced collagen development. Hence, MAC-PEG-DMA (PDGF-AA) exhibits significant promise as a dressing for enhancing the process of wound healing.

**FIGURE 6:**
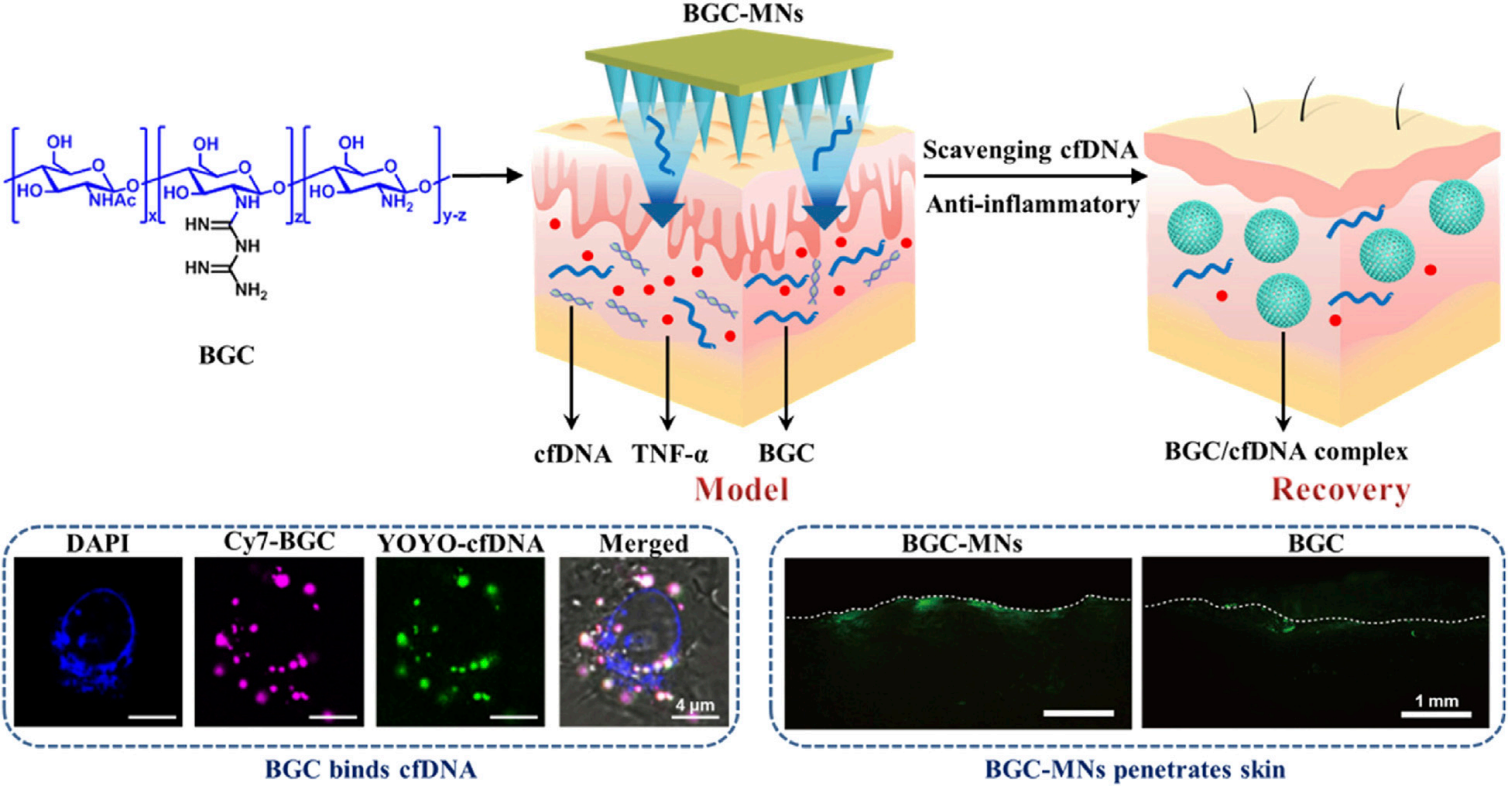
Cationic biguanide chitosan microneedles (BGC-MNs) were created. BGC-MNs penetrate the skin painlessly and efficiently scavenge cfDNA to treat psoriasis. BGC binds cfDNA since they are both in cells. FITC-BGC diffusion in the skin shows that microneedles penetrate better than coating. Reproduced with permission from the ref. Liu Y. et al. (2024), Copyright 2024, Elsevier. (This work is licensed under CC BY-NC-ND 4.0. To view a copy of this license, visit https://creativecommons.org/licenses/by-nc-nd/4.0/).

Psoriasis may be brought on by elevated amounts of cell-free DNA (cfDNA). Psoriasis treatments now in use have disadvantages including skin irritation, reduction of natural immunity, and trouble crossing the skin barrier. To solve these problems, Liu and colleagues created biguanide chitosan microneedles (BGC-MNs), which eliminate cfDNA from the dermis (Liu Z. H. et al., 2024) (Figure 6). Regarding binding DNA, compatibility with living things, and inflammation reduction, the chitosan containing 20% bisguanidine (BGC2) performed the best overall. BGC2 was shown *in vitro* to efficiently remove cfDNA and reduce the synthesis of inflammatory factors. Favorable mechanical and solubility characteristics were shown by BGC-MNs derived from BGC2. In animal experiments on psoriasis-affected mice, BGC-MNs reduced dermal inflammation, eliminated cfDNA, and promoted skin healing. These results imply that by scavenging cfDNA and causing anti-inflammatory effects, BGC-MNs provide a viable new strategy for treating psoriasis.

Current challenges in chitin and chitosan research involve enhancing extraction and production methods to boost yield and purity while lowering costs. Additionally, efforts are underway to develop more efficient deacetylation processes for chitosan, and to enhance the reproducibility and standardization of chitin-derived products. A deeper understanding of the structure-function relationships of these biopolymers and their derivatives is also necessary. The main problems with chitin and chitosan in tissue engineering are that they don't always work the same way from batch to batch, some forms don't have enough mechanical strength, and some people may have immune reactions to them. Despite these challenges, the future for chitin and chitosan remains promising. Their unique properties, biocompatibility, and biodegradability make them appealing for various applications, including biomedicine, agriculture, water treatment, and food packaging. Ongoing research is focusing on creating novel chitin-based materials with enhanced properties, discovering new applications in tissue engineering and drug delivery, and utilizing chitosan to develop functional nanomaterials. In the coming years, chitin and chitosan, as sustainable and eco-friendly alternatives to synthetic polymers, can play an increasingly important role in the advancement of green technologies and circular economy solutions.

### 2.3 Cellulose

Natural polysaccharides are abundant in nature and consist of polymer complexes formed by the linkage of numerous monosaccharide molecules through glycosidic linkages. Biopolymers including alginate, cellulose, and hyaluronic acid possess exceptional qualities that make them ideal for usage as bioinks. These qualities include outstanding biocompatibility and rheological capabilities.

Cellulose is a naturally occurring polymer that is biocompatible and biodegradable. It does not elicit strong immune reactions when implanted in the human body, making it an appropriate material for tissue engineering scaffolds (Hickey and Pelling, 2019). Cellulose exhibits excellent mechanical robustness and pliability. Chemical or physical alterations can be made to further fine-tune its mechanical characteristics to fulfill the specific demands of different biological applications. For instance, cross-linking can be employed to augment its mechanical stability. Cellulose has a molecular structure that enables various chemical changes, such as carboxymethylation and acetylation. These alterations could modify the solubility, bioactivity, and breakdown rate of cellulose, hence broadening its potential applications in tissue engineering (Aziz et al., 2022). Cellulose can undergo numerous forms of processing, such as being transformed into fibers, sponges, membranes, or hydrogels. These varied forms are well-suited for a variety of tissue engineering applications (Zainal et al., 2021; Abdelhamid and Mathew, 2022). For example, cellulose-based sponges can be utilized as frameworks for skin regeneration (Tudoroiu et al., 2021), whereas cellulose membranes can be employed for wound dressing (de Amorim et al., 2022). Cellulose scaffolds have the potential to enhance cell adhesion, stimulate cell proliferation, and facilitate cell differentiation through surface modification. By grafting peptide sequences or growth factors onto the surface of cellulose, it is possible to enhance cell activity on the scaffold. Research has demonstrated that scaffolds made from cellulose could facilitate the development of many types of cells, such as osteoblasts, chondrocytes, and skin cells (Iravani and Varma, 2022). This suggests that cellulose-based scaffolds have promising applications in tissue engineering for bone, cartilage, and skin. Table 3 encapsulates recent research on cellulose and its implications in tissue engineering and regenerative medicine.

**TABLE 3:** Recent studies on cellulose and their outcomes.

Cellulose type	Research topic	Research outcome	References
Cellulose	Cellulose-based flexible electronics	Using cellulose nanofibrils as a substrate with biological tissues, emphasizing its potential in developing wearable sensors, supercapacitors, and other healthcare-related devices	Saleh et al. (2024)
Cellulose nanocrystals	Cellulose nanocrystals for drug delivery	Demonstrated the potential of cellulose nanocrystals as drug carriers for controlled release and targeted delivery	Li et al. (2024c)
Bacterial cellulose	Bacterial cellulose for wound healing	Developed bacterial cellulose-based wound dressings with improved healing properties and antimicrobial activity	Gouripriya et al. (2024), Deng et al. (2024), Khan et al. (2024), Li et al. (2024b)
Cellulose nanofibers	Cellulose nanofibers for 3D printing	Developed cellulose nanofiber-based inks for 3D printing of complex structures with improved mechanical properties	Iglesias-Mejuto et al. (2024)
Cellulose	Cellulose-based smart textiles for Multi-protection	Created cellulose-based smart textiles with flexible multifunctional properties	Yu et al. (2024)

Among all types of cellulose, bacterial cellulose (BC) is a three-dimensional carbohydrate polymer produced by bacteria. BC exhibits strong biocompatibility and holds promise as a suitable material for use as a substrate in the development of artificial skin. BC aids in wound healing by creating a humid environment. BC is an excellent choice for producing synthetic skin due to its affordability and uncomplicated manufacturing procedure. Liu et al. created an artificial skin using BC sandwiched between photosensitizers (PSs) and functionalized live cells (Liu X. et al., 2024). The researchers created a photosensitizer with strong ROS production and modified it with β-D-glucose to make glucose-modified PS (TBG). In a traditional static culture, they progressively fed TBG and azide-modified glucose as carbon substrates, resulting in a structured BC with a PS layer and an azide-rich layer on each side. Then, using an alkynyl group, they created VEGF-transfected human umbilical vein endothelial cells (HV) and grafted them onto the azide-rich BC layer via a bio-orthogonal reaction to form an antibacterial and live cell-based therapeutic artificial skin (HV@BC@TBG). The exterior layer of the artificial skin contains a TBG layer that can generate reactive oxygen species (ROS) when exposed to light. This ROS production is effective in eliminating harmful bacteria and preventing the entry of foreign bacteria into wound sites. The HV layer, located on the inner side of the artificial skin near the diabetic wound sites, acts as a biological factory to produce VEGF directly at the site. This process enhances the formation of new blood vessels and encourages the growth and movement of cells that line the inner surface of blood vessels, so expediting the healing of wounds. The findings demonstrated that the biosynthetic HV@BC@TBG exhibits exceptional bactericidal properties and accelerates wound healing. This demonstrates the considerable capacity of the material to function as an advanced artificial substance for the treatment of long-lasting wounds. This indicates its promise as a high-performance artificial material for chronic wounds.

Dang et al. made CPPFe@TA hydrogels using CMC, PEI, PAM, and Fe^3+^ which self-polymerized *in situ* via TA@Fe^3+^ (Dang et al., 2024). CPPFe@TA hydrogels had many useful properties, including self-starting, time-changing, mechanical performance, self-healing, high adhesion, strong antibacterial activity, good conductivity, and biocompatibility. Hydrogel dressings exposed to near-infrared light revealed photothermal antibacterial activity in a mouse model with a full-thickness skin defect and illness. This accelerated wound healing. Using CMC’s conductive and thermosensitive characteristics, the hydrogel might become a flexible skin-integrated biosensing device. This device may monitor physiological signals and temperature changes in real time, providing early infection warning signals, severity evaluation indicators, and vital clinical information for rehabilitation training. For practical use, the hydrogel’s frost resistance and excessive water retention need development. Monitoring human physiological signals should be made more repeatable and practicable through study. Overall, the CPPFe@TA hydrogel shows great potential for wound treatment and smart healthcare. It expands bioelectronics-regenerative wound healing applications.

In summary, cellulose’s biocompatibility, mechanical properties, and versatility make it a promising material for tissue engineering. Its ability to be processed into various forms, modified for improved biological interactions, and combined with other biomaterials allows for the creation of tailored scaffolds for specific tissue engineering applications. As research advances, cellulose-based materials are expected to play an increasingly important role in regenerative medicine and tissue repair. Nevertheless, cellulose in the field of tissue engineering presents various limitations and obstacles. Cellulose-based scaffolds may exhibit inadequate cell attachment and spread due to the absence of inherent cell-binding sites in cellulose. In order to enhance the interactions between cells and materials, it may be necessary to chemically modify or functionalize them with cell-adhesive peptides. Although cellulose is inherently biocompatible, any remaining impurities from its extraction and processing, such as those derived from plant sources, have the potential to elicit immune reactions. In order to reduce the likelihood of an immune response, it is crucial to conduct a meticulous purification process. Cellulose-based scaffolds may not consistently exhibit the same mechanical characteristics as the original tissue they are intended to replace. Cellulose exhibits a significant tensile strength, while its compressive strength is comparatively lower than that of bone. The mechanical properties of composites can be adjusted by incorporating cellulose with other materials. The rate at which cellulose degrades in living organisms is relatively sluggish when compared to certain other natural polymers commonly employed in tissue engineering. This can pose a difficulty for applications in which there is a need for quicker breakdown of the scaffold and its replacement with new tissue. If necessary, cellulose degradation can be expedited through chemical modification. Cellulose can undergo degradation or alteration of its properties when exposed to common sterilization methods, such as heat and radiation. It may be necessary to develop specific techniques for processing and sterilizing cellulose in order to preserve its structural integrity for use in clinical applications. Notwithstanding these difficulties, the beneficial characteristics of cellulose persistently motivate research efforts to overcome these restrictions for tissue engineering purposes. Promising strategies such as composites, chemical modification, and new processing methods offer potential for fully utilizing the abundant natural polymer.

Cellulose research faces several challenges, particularly in developing more efficient and environmentally friendly methods for extracting and processing cellulose from non-wood sources. There is a need to improve the compatibility of cellulose with other materials for composite applications and to enhance its properties for specific uses. Additionally, scaling up the production of advanced cellulose-based materials while maintaining cost-effectiveness presents a significant hurdle. Despite these challenges, cellulose holds enormous promise for the future. Its abundance, renewability, and biodegradability make it an attractive alternative to petroleum-based materials. Emerging opportunities include the development of high-performance nanocellulose materials for applications in electronics, energy storage, and biomedical fields. Researchers are also exploring cellulose-based smart materials with responsive properties for use in sensors and actuators.

### 2.4 Hyaluronic acids

Hyaluronic acid (HA) is a natural glycosaminoglycan present in the extracellular matrix of connective tissues in the body (Braccini et al., 2024). The compound is a linear polysaccharide consisting of a repeating unit of D-glucuronic acid and N-acetyl-D-glucosamine (Iaconisi et al., 2023). HA exhibits excellent biocompatibility, as it does not provoke an immunological reaction upon implantation in the body (Dovedytis et al., 2020). Furthermore, HA undergoes natural degradation via hyaluronidases present in the body (Garantziotis and Savani, 2019). Chemical modification can regulate the rate at which HA breaks down, allowing for the creation of scaffolds with specific degradation patterns that match the pace of new tissue development. Tissue engineering constructions that are intended to integrate with host tissues can be advantageous by utilizing the distinctive viscoelastic and hygroscopic characteristics of HA. Hydrogel scaffolds used in 3D cell culture and tissue regeneration can incorporate HA. HA hydrogels imitate the natural extracellular matrix and create a favorable environment for cell attachment, growth, and specialization (Yasin et al., 2022).

Hyaluronic acid binds to cell surface receptors, including as CD44 and RHAMM, to control a range of cellular activities, including cell movement, growth, and specialization. These interactions can be harnessed to manipulate cell activity in tissue engineering applications. HA can be altered using growth factors, cytokines, or pharmaceuticals to develop delivery systems that enable the regulated release of bioactive substances, which facilitate the regeneration of tissues (Misra et al., 2015; Leng et al., 2019; Nicolas et al., 2020). HA based-biomaterials have been extensively researched for a range of tissue engineering purposes, such as cartilage tissue engineering, skin wound healing, bone tissue engineering, and brain tissue engineering.

Hyaluronic acid has unique physicochemical features that improve molecular delivery and matrix interactions, both of which are essential for chondrocyte survival and function. Shim et al. utilized HA microparticles as a conduit for integrating nutrients into the spheroid. The primary objective of this work is to examine if the inclusion of HA particles within chondrocyte spheroids can improve their viability and capacity to generate cartilage. The incorporation of HA microparticles into spheroid formations is a promising and revolutionary approach to regenerate cartilage. The innovative combination of HA and spheroid culture presents a promising opportunity for the development of more efficient treatments for osteoarthritis (Shim et al., 2024). Recent research on hyaluronic acids and how they work in tissue engineering and regenerative medicine is summed up in Table 4.

**TABLE 4:** Recent studies on Hyaluronic acid and their outcomes.

Hyaluronic acid type	Research topic	Research outcome	References
Hyaluronic acid	HA-based hydrogels for cartilage regeneration	HA hydrogels that promote cartilage regeneration and reduce osteoarthritis progression	Zhang et al. (2024b)
Hyaluronic acid	HA nanoparticles for bone tissue regeneration	Develop HA based multifunctional nanoparticles by inheriting the membrane functions of the source cells, possess prolonged circulation and specific localization at the inflamed sites	Liu et al. (2024b)
Hyaluronic acid	HA-based scaffolds for wound healing	Developed HA-based scaffolds with enhanced wound healing properties	Graça et al. (2020), Alven and Aderibigbe (2021), Jung et al. (2024)
Hyaluronic acid	HA-based drug delivery systems for ocular diseases	Developed HA-based nanocarriers for improved drug delivery to the eye, enhancing treatment of ocular diseases	Zhang et al. (2021), Tsung et al. (2023)
Hyaluronic acid	HA-based bioinks for 3D bioprinting	Developed HA-based bioinks with tunable properties for 3D bioprinting of tissue constructs	Kim et al. (2024b), Li et al. (2024a), Tavakoli et al. (2024), Tripathi et al. (2024)

**FIGURE 7:**
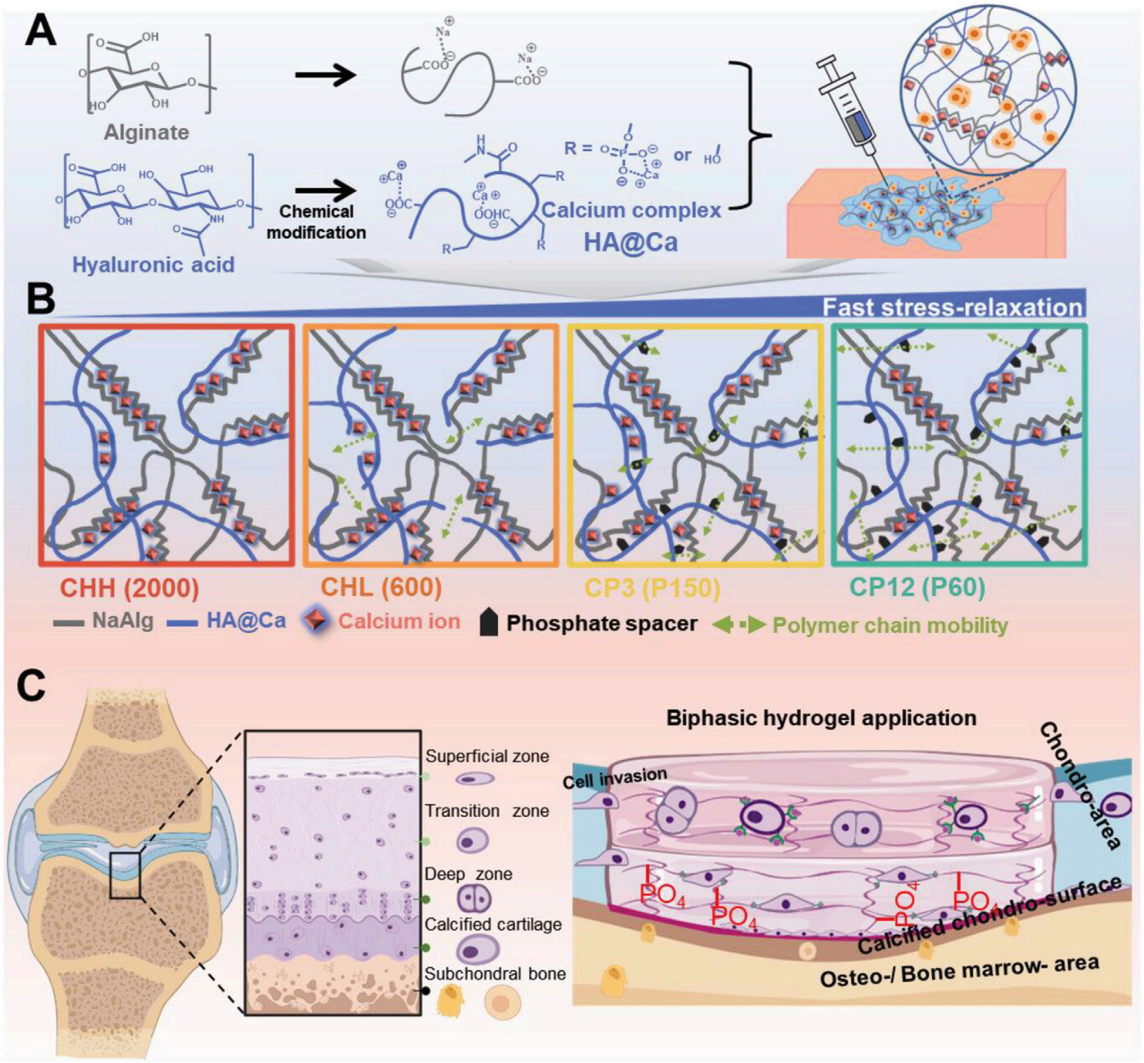
Hydrogel preparation and remodeling. **(A)** Chemical structure of HA@Ca and injectable hydrogels, displaying self-healing and mechanical adjustability (stress relaxation, shear thinning). **(B)** Schematic of the improved remodeling hydrogel mechanism due to molecular weight and phosphate spacers increasing polymer chain mobility. **(C)** Schematic and use of the hyaluronic acid-based biphasic hydrogel for selective hyaline and calcified cartilage regeneration via CD44 receptors. Reproduced with permission from the ref. Kim H.-S. et al. (2024), Copyright 2024, John Wiley and Sons. (This work is licensed under CC BY 4.0. To view a copy of this license, visit https://creativecommons.org/licenses/by/4.0/).

Kim et al. address the creation of a novel hydrogel with remodeling properties by combining hyaluronic acid and calcium (HA@Ca). By adding HA@Ca, the researchers were able to change the molecular weight and degree of hyaluronic acid phosphorylation, giving them control over the hydrogel’s remodeling capabilities. By varying the type and amount of alginate and HA@Ca, the researchers were able to gradually alter the hydrogel’s stiffness and capacity to relax under stress. This was accomplished by adjusting the fluidity of the polymer chains. The researchers conducted a thorough investigation into the ideal circumstances for cartilage regeneration under extremely limited stiffness settings by fine-tuning the stress relaxation duration and phosphorylation level. The findings revealed that faster stress release in the hydrogel considerably increased the development of cartilage substrates in chondrocytes. However, the influence on chondrocytes differed depending on the hydrogel’s composition. As a result, the anticipated regeneration outcomes must be taken into account while selecting a composition for cartilage and stem cell cultures. The successful regeneration of chondrocytes and calcified cartilage using HA@Ca demonstrates the possibility for more effective and tailored cartilage regeneration techniques. Developing a hydrogel system that can regulate these physicochemical qualities could serve as a tailored culture platform for cells with specific characteristics. Overall, this discovery has great potential in the field of cartilage regeneration since it introduces HA-based hydrogels with custom-remodeling capabilities. The capacity of hydrogels to precisely tune their mechanical and stress-relieving qualities opens up new possibilities for stem cell therapy and tissue regeneration (Kim H.-S. et al., 2024) (Figure 7).

In summary, hyaluronic acid is a versatile biomaterial with unique properties that make it well-suited for various tissue engineering applications. Its biocompatibility, biodegradability, and ability to interact with cells and deliver bioactive molecules have led to its widespread use in the development of scaffolds and delivery systems for regenerative medicine. There exist certain limitations and obstacles linked to the utilization of HA in the field of tissue engineering. Enzymes known as hyaluronidases rapidly break down HA, which may restrict its ability to maintain long-term stability and mechanical strength as a scaffold material. The mechanical strength and stiffness of native HA are relatively low, which limits its suitability for load-bearing tissue engineering applications. HA derived from animals or produced by microbes can exhibit variations in molecular weight, purity, and chemical composition, posing challenges in achieving consistent production of HA-based structures. HA lacks the identical cell adhesion motifs found in other extracellular matrix proteins. This can impede the adhesion and proliferation of cells on HA scaffolds. While animal-derived HA is generally biocompatible, it can potentially contain impurities or contaminants that may trigger an immune response, leading to inflammation or rejection of the tissue engineering construct. Scientists are currently developing methods to overcome these constraints, including chemical alteration, merging HA with other biomaterials, and employing recombinant or synthetic HA. As these strategies progress, the potential of HA in tissue engineering is anticipated to increase.

Hyaluronic acid research faces several challenges. One major issue is the need for more cost-effective and sustainable production methods, as current processes, which rely on bacterial fermentation or animal extraction, can be expensive and raise ethical concerns. Additionally, improving the stability and longevity of HA-based products, particularly in biomedical applications, remains critical. Another ongoing challenge is enhancing the targeting and controlled release capabilities of HA in drug delivery systems. Despite these hurdles, the prospects for HA are highly promising. Its unique biocompatibility, biodegradability, and versatility make it invaluable across various fields. In cosmetics and aesthetics, HA remains a cornerstone ingredient, with ongoing research focusing on developing more effective formulations and delivery methods. In the medical field, HA shows enormous potential in tissue engineering and regenerative medicine, and it serves as a platform for advanced drug delivery systems. Emerging applications include its use in wound healing, osteoarthritis treatment, and as a biomarker for various diseases. The development of HA-based biomaterials with enhanced properties such as improved mechanical strength or antimicrobial activity opens up new possibilities in biomedical engineering. As research advances, we expect HA to play an increasingly important role in personalized medicine, nanomedicine, and the development of smart, responsive biomaterials.

### 2.5 Alginates

Alginates are naturally occurring polysaccharides derived from brown algae. Their biocompatibility, low toxicity, and ability to form hydrogels in mild conditions have led to their widespread use in tissue engineering applications (Zhang Q. et al., 2024). Alginates are highly biocompatible and generally tolerated by the human body. They cause minimal immune response and inflammation when implanted, making them suitable for a variety of tissue engineering applications. Ionic crosslinking allows alginates to form hydrogels when exposed to divalent cations such as calcium (Ca^2+^). Cells and bioactive molecules can be encapsulated in a 3D matrix that replicates the natural extracellular environment. The physical and chemical properties of alginate hydrogels can be tailored by adjusting the molecular weight, composition (guluronic acid to mannuronic acid ratio), and concentration of the alginate, as well as the type and concentration of crosslinking ions. Alginate hydrogels can encapsulate a variety of cell types for regenerative medicine applications, including stem cells, chondrocytes, and islet cells. The hydrogel offers a 3D environment that promotes cell survival, proliferation, and differentiation. Freeze-drying, 3D printing, and electrospinning are all methods for converting alginates into porous scaffolds. Tissue regeneration can benefit from these scaffolds, which provide a temporary matrix for cell attachment, migration, and new tissue formation. Furthermore, alginate hydrogels can be used as delivery vehicles for controlled release of drugs, proteins, or growth factors to promote tissue regeneration and treat local diseases. Alginates have been studied for their potential use in tissue engineering applications such as cartilage, bone, skin, liver, and pancreatic regeneration. Table 5 encapsulates recent research on alginates and their results in tissue engineering and regenerative medicine.

**TABLE 5:** Recent studies on Alginates and their outcomes.

Alginates Type	Research topic	Research outcome	References
Alginate	Alginate-based hydrogels for wound healing	Developed photo-crosslinking method to prepare multifunctional sodium alginate-based hydrogel dressings for effective wound healing/Bio-based nanoparticles were created and added to 3D printing alginate-based ink. Subsequently, a 3D structural extrusion printer was employed to create the porous hydrogel-based wound dressings	Zhang et al. (2024a), Ashtariyan et al. (2024)
Alginate	Alginate nanoparticles for drug delivery	Created alginate-based encapsulation systems for targeted drug delivery to realize tailored drug loading, enhanced stability, and sustained release kinetics	Lai et al. (2024), Rajapaksha et al. (2024)
Alginate	3D bioprinting with alginate bioinks	Developed alginate-based bioinks with improved printability and cell viability for 3D bioprinting of tissue constructs	Chrungoo et al. (2024)
Alginate	Alginate-based materials for bone tissue engineering	Created composite scaffolds of alginate for enhanced bone regeneration/promote chondrocyte growth in 2D and 3D cultures	Chen et al. (2024), Hang et al. (2024)
Alginate	Alginate microencapsulation for cell therapy	Developed alginate-based hydrogels for microencapsulating targeted towards cell therapy	Suzuki et al. (2023), Ambrosino et al. (2024)
Alginate	Alginate-based scaffolds for cardiac tissue engineering	Alginate-based hydrogel loaded with mitochondria accelerated angiogenesis in a rat model with acute myocardial infarction/Apply 3D printing to create cardiac constructions with a new angular structure that mimics heart architecture from alginate (Alg) and gelatin (Gel)	Hassanpour et al. (2024), Ketabat et al. (2023)

Zhang et al. developed injectable SA-X (X = Ca^2+^, Cu^2+^, Co^2+^, Mn^2+^, Zn^2+^, Ni^2+^, Sr^2+^, and Fe^3+^) hydrogels by employing a uniform-unsaturated crosslinking strategy. Metal ions are coordinated with the guluronate moieties of SA molecules through vigorous stirring. This technique avoids the excessive local crosslinking and injectability loss that can occur with traditional dropping and soaking methods. The injectability of SA hydrogels can be altered by varying the concentration of metal ions. Furthermore, multiple metal ions can now be used to crosslink hydrogels, expanding their functions. The SA hydrogels can serve as a general platform for developing composites for targeted drug delivery, tissue repair, and wound infection management. The study showed that injectable SA-0.2%Cu hydrogel can promote the healing of full-thickness skin, indicating its potential for wound infection treatment. This study not only presents a simple and versatile strategy for producing various SA injectable hydrogels at a low cost, but it also emphasizes their potential applications in drug delivery, bone cement formation, and wound infection treatment. These findings demonstrate the hydrogels’ significant potential in biomedical applications and clinical use (Zhang Q. et al., 2024).

The researchers used oxidized sodium alginate (OA), a controlled oxidation derivative with a high affinity for dECM (decellularized extracellular matrix) due to its aldehyde groups. This contributes to the retention of growth factors and other molecules. In addition, they integrated tricalcium silicate (TCS) into a scaffold made of polycaprolactone (PCL) for the purpose of reconstructing bone defects. The study utilized 3D printing to create scaffolds made of polycaprolactone (PCL) with a maximum of 30% tricalcium silicate (TCS). These scaffolds were then modified by incorporating decellularized bone matrix-oxidized sodium alginate (DBM-OA). The findings demonstrated that the inclusion of 20% TCS resulted in a substantial enhancement of the compressive modulus, which increased by a factor of 4.5. Similarly, the yield strength experienced a significant increase of 12-fold, while the toughness showed a remarkable enhancement of 15-fold when compared to pure PCL. Furthermore, the samples containing TCS exhibited the development of crystalline phases with a Ca/P ratio resembling that of hydroxyapatite (1.67). TCS was found to enhance the biocompatibility of PCL-based scaffolds, as demonstrated in cellular experiments. After a period of 7 days, the scaffolds that had been altered with DBM (demineralized bone matrix) and 20% TCS (tricalcium phosphate) exhibited a significant increase in ALP (alkaline phosphatase) activity compared to pure PCL (polycaprolactone) scaffolds. Specifically, the modified scaffolds demonstrated eight times higher ALP activity in placenta-derived mesenchymal stem/stromal cells (P-MSCs) than the unmodified PCL scaffolds. The study indicates that the inclusion of TCS and DBM-OA in 3D-printed PCL-based scaffolds enhanced their mechanical properties, bioactivity, hydrophilicity, and ability to promote bone formation. Therefore, they are an excellent option for applications in bone tissue engineering (Menarbazari et al., 2024).

Alginate is a highly adaptable and biocompatible substance that has been widely employed in tissue engineering for tasks such as cell encapsulation, scaffold creation, and drug administration. The tunable characteristics and capacity to create hydrogels under gentle conditions render it a compelling option for diverse tissue-specific applications in the field of regenerative medicine. Alginate poses several challenges in tissue engineering, including restricted cell adhesion, the requirement for functionalization to enhance cell-material interactions, and the absence of inherent biodegradability. Nevertheless, these constraints can be surmounted by altering alginate through the addition of cell-adhesive peptides or by amalgamating it with other biodegradable polymers.

Alginate research faces several challenges, including the need for consistent and controlled production methods to ensure uniform quality, as natural sources can vary significantly. Improving the mechanical properties and stability of alginate-based materials, particularly under physiological conditions, is a key issue for biomedical applications. Additionally, enhancing the functionalization of alginate to expand its uses and developing more efficient crosslinking methods are ongoing challenges. Despite these hurdles, the prospects for alginate are promising. Its biocompatibility, biodegradability, and versatility make it attractive for various applications. In the biomedical field, alginate shows enormous potential for tissue engineering, drug delivery systems, and wound healing. Current research focuses on developing advanced alginate-based hydrogels with improved properties for cell encapsulation and 3D bioprinting. In the food industry, alginate continues to be valuable as a thickening and stabilizing agent, with new applications emerging in functional foods and nutraceuticals. Researchers are also exploring environmental applications, such as using alginate for heavy metal removal in water treatment and as a biodegradable alternative to synthetic plastics. Moreover, the development of alginate-based smart materials with responsive properties opens up possibilities for sensors and actuators. As research advances, we anticipate that alginate will significantly contribute to the development of sustainable, bio-based solutions in various industries, thereby advancing the fields of green chemistry and materials science.

## 3 Summary and outlooks

Natural polymers have become a fundamental component in the field of tissue engineering due to their inherent biocompatibility, biodegradability, and bioavailability. These characteristics make them optimal candidates for constructing scaffolds capable of facilitating cell proliferation and tissue regeneration. These materials can serve as structures that support the growth, multiplication, and specialization of cells, ultimately aiding in the restoration and healing of damaged or diseased tissues. Natural polymers possess a wide range of applications, as they can be used either alone or in conjunction with other materials, enabling researchers to precisely adjust the mechanical characteristics of scaffolds to meet the distinct needs of various tissues. The utilization of natural polymers in tissue engineering holds immense potential, encompassing a wide range of applications such as the regeneration of bone and cartilage, repair of soft tissue, and reconstruction of organs. By mimicking the extracellular matrix, they create a recognizable environment for cells, which enhances their ability to adhere, proliferate, and differentiate. The use of biomimetic strategies is crucial for achieving effective tissue integration and regeneration. As our understanding of the interactions between natural polymers and host tissues grows, we can expect significant advancements in tissue engineering. Some promising areas of research include: (1) Smart materials, which involve studying polymers that can respond to physiological stimuli and adapt their properties accordingly; (2) *In situ* tissue engineering, which focuses on developing injectable polymer systems that can directly form scaffolds within the body; (3) Hybrid approaches, which combine natural polymers with synthetic materials or inorganic components to enhance scaffold properties; and (4) Immunomodulatory scaffolds, which involve designing materials that can modulate the immune response to promote tissue regeneration and minimize rejection.

However, there are several challenges associated with using natural polymers in tissue engineering. One of the main issues is vascularization, which is the process of creating blood vessels in a tissue or organ. Achieving adequate vascularization is a significant challenge in tissue engineering, especially for large implants. If the scaffold lacks sufficient blood supply, it can lead to a shortage of oxygen and nutrients reaching the cells, potentially causing necrosis. Scientists are exploring various methods to promote blood vessel growth, such as incorporating angiogenic factors into the scaffolds or using co-culture systems that combine endothelial cells with other cell types. The controlled release of bioactive molecules is another important consideration. While incorporating bioactive molecules into polymeric scaffolds has the potential to enhance tissue regeneration, effectively managing their release is a significant challenge. An ideal scaffold should enable a sustained and controlled release of growth factors and other bioactive agents to guide tissue formation. Researchers are investigating advanced delivery strategies, such as using nanoparticles or stimuli-responsive polymers, to address this issue. However, the use of bioactive molecules in tissue engineering raises important concerns regarding safety and efficacy. It is essential to thoroughly evaluate the long-term effects of these molecules on tissue regeneration and their potential side effects. Additionally, determining the optimal concentration and delivery method for each bioactive agent is crucial for achieving the desired therapeutic outcome. Extensive preclinical studies and clinical trials are necessary to establish standardized protocols. Furthermore, traditional fabrication methods often face challenges in producing scaffolds with precise control over pore size, architecture, and surface properties. These factors are critical for facilitating cell infiltration, nutrient transport, and maintaining mechanical integrity. To overcome these limitations, researchers are exploring advanced manufacturing technologies, such as 3D printing and electrospinning, which enable the fabrication of complex and biomimetic scaffolds.

In summary, while natural polymers show promise in tissue engineering, overcoming current challenges requires a multidisciplinary approach. By combining advancements in polymer science, cell biology, and manufacturing technologies, we can fully utilize the natural polymers’ inherent capabilities in regenerative medicine. In the future, progress in bioprinting and other advanced fabrication techniques, coupled with better understanding of cell-material interactions, will allow for the development of more effective and customized tissue engineering solutions.
